# Endophytic Bacteria Improve Plant Growth, Symbiotic Performance of Chickpea (*Cicer arietinum* L.) and Induce Suppression of Root Rot Caused by *Fusarium solani* under Salt Stress

**DOI:** 10.3389/fmicb.2017.01887

**Published:** 2017-09-28

**Authors:** Dilfuza Egamberdieva, Stephan J. Wirth, Vyacheslav V. Shurigin, Abeer Hashem, Elsayed F. Abd_Allah

**Affiliations:** ^1^Leibniz Centre for Agricultural Landscape Research, Institute of Landscape Biogeochemistry, Müncheberg, Germany; ^2^Faculty of Biology, National University of Uzbekistan, Tashkent, Uzbekistan; ^3^Botany and Microbiology Department, College of Science, King Saud University, Riyadh, Saudi Arabia; ^4^Mycology and Plant Disease Survey Department, Plant Pathology Research Institute, Giza, Egypt; ^5^Plant Production Department, College of Food and Agricultural Sciences, King Saud University, Riyadh, Saudi Arabia

**Keywords:** chickpea, salinity, endophytes, rhizobia, symbioses, root rot

## Abstract

Salinity causes disturbance in symbiotic performance of plants, and increases susceptibility of plants to soil-borne pathogens. Endophytic bacteria are an essential determinant of cross-tolerance to biotic and abiotic stresses in plants. The aim of this study was to isolate non–rhizobial endophytic bacteria from the root nodules of chickpea (*Cicer arietinum* L.), and to assess their ability to improve plant growth and symbiotic performance, and to control root rot in chickpea under saline soil conditions. A total of 40 bacterial isolates from internal root tissues of chickpea grown in salinated soil were isolated. Four bacterial isolates, namely *Bacillus cereus* NUU1*, Achromobacter xylosoxidans* NUU2, *Bacillus thuringiensis* NUU3, and *Bacillus subtilis* NUU4 colonizing root tissue demonstrated plant beneficial traits and/or antagonistic activity against *F. solani* and thus were characterized in more detail. The strain *B. subtilis* NUU4 proved significant plant growth promotion capabilities, improved symbiotic performance of host plant with rhizobia, and promoted yield under saline soil as compared to untreated control plants under field conditions. A combined inoculation of chickpea with *M. ciceri* IC53 and *B. subtilis* NUU4 decreased H_2_O_2_ concentrations and increased proline contents compared to the un-inoculated plants indicating an alleviation of adverse effects of salt stress. Furthermore, the bacterial isolate was capable to reduce the infection rate of root rot in chickpea caused by *F. solani*. This is the first report of *F. solani* causing root rot of chickpea in a salinated soil of Uzbekistan. Our findings demonstrated that the endophytic *B. subtilis* strain NUU4 provides high potentials as a stimulator for plant growth and as biological control agent of chickpea root rot under saline soil conditions. These multiple relationships could provide promising practical approaches to increase the productivity of legumes under salt stress.

## Introduction

Legumes are highly important crops in human and animal nutrition and are grown globally under a wide range of agro-climatic conditions as a cash crop and as a source of nitrogen assimilation via nitrogen fixation (Lüscher et al., 2011; Nyfeler et al., 2011). Legumes form important symbiotic relationships with rhizobia and are known as the most efficient system for biological nitrogen fixation (BNF) (Molla et al., 2001; Egamberdieva et al., 2013, 2016a; Santi et al., 2013). Numerous studies have shown that the symbiotic relationship between legumes and their rhizobia are susceptible to abiotic factors such as salinity, drought, and soil temperature, which can cause a failure in the infection and nodulation process (Slattery et al., 2001; Bouhmouch et al., 2005). Salt stress inhibits plant growth, nutrient uptake, and increases susceptibility of plants to soil-borne pathogens (Egamberdieva et al., 2011; Ahmad et al., 2015; Hashem et al., 2016). The susceptibility of plants to infection by soil borne pathogens was increased by salt stress, e.g., tomato root rot caused by *Fusarium oxysporum* f. sp. *radicis-lycopersici* (Triky-Dotan et al., 2005), and cucumber root rot caused by *Fusarium solani* (Egamberdieva et al., 2011). Nevertheless, microbes associated with a plant can have beneficial interactions, providing its partner organism biologically active compounds necessary for survival and proliferation (Marschner et al., 2001; Mercado-Blanco et al., 2004).

The interest in endophytic bacteria has increased, as they colonize the internal tissues of their host plants and improve plant tolerance to various abiotic stress factors and can protect plants from various pathogenic microbes (Malfanova et al., 2011; Hashem et al., 2016). Endophytic bacteria were found in different plants including crops, aromatic and medicinal plants, halophytes etc. (Azarias Guimarães et al., 2012; Sharma et al., 2012; Egamberdieva et al., 2017). Mutualistic associations between root associated microbes and plants bring benefits to the plant through an increased nutrient acquisition, altered metabolic interactions among the partners, alleviation of salt stress and improved symbiotic performance of legumes. The endophytic lifestyle may directly or indirectly assist during the infection and colonization processes of the rhizobium-host association and are coordinately involved in the adaptation of plants to stress tolerance (Hashem et al., 2016). The endophytes which effectively colonize plant tissue could be even more beneficial in a co-inoculation with rhizobia under salt stress as shown by previous work (Egamberdieva et al., 2016a). In addition, endophytic bacteria colonize root tissues and are capable to protect their hosts against invasion and damage by soil-borne pathogens (Mercado-Blanco et al., 2004; Rybakova et al., 2016). Endophytic bacteria colonizing internal plant tissue benefit plants using various traits, including synthesis of plant growth regulators (Beneduzi et al., 2012), osmoprotectants, exopolysaccharides (Berg et al., 2013), antifungal metabolites (Gond et al., 2015), and the modulation of plant physio-biochemical constituents (Hashem et al., 2016). However, despite the importance of the endophyte-plant relationship, our knowledge on the interactions between legumes, endophytes, and pathogens under hostile environmental conditions is still rather limited. Chickpea (*Cicer arietinum* L.) is a major food legume crop and an important source of protein in many countries, however its production is restricted by soil borne diseases and abiotic stress (Graham and Vance, 2003). A black root rot caused by *F. solani* is amongst the most serious fungal diseases of chickpea (Andrabi et al., 2011; Cabral et al., 2016).

The aims of the present study were to: (i) isolate and identify endophytic bacteria from the root nodule of chickpea with potential colonization patterns, (ii) assess their efficiency in improvement of plant growth, symbiotic performance of host plant, and alleviating salt stress, (iii) determine their biological control capability against *Fusarium* root rot on chickpea. Thus, our study intends to provide valuable information about interactions among rhizobia, endophytes, pathogens, and hosts under salt stress conditions.

## Materials and methods

### Isolation of bacteria from chickpea nodules

Samples of healthy chickpea plants were collected from a field in the Syrdarya province of Uzbekistan, which is considered a salt affected region with summer daytime temperatures between 38 and 40°C. Soil is loamy sand and characterization was as follows: 43 ± 9 g sand kg^−1^, 708 ± 12 g silt kg^−1^, and 250 ± 13 g clay kg^−1^, and had a cation exchange capacity of 23.6 ± 1 cmol kg^−1^, with an exchangeable Na percentage of 4.41 and a Na absorption ratio of 0.32 (Egamberdieva et al., 2010). Electrical conductivity (EC) values of the saline soil were 7.2 dS m^−1^. The organic matter content of the soil was 0.69% with total C, 2.5%; total N, 0.1%; Ca, 63.5 g/kg; Mg, 20.7 g/kg; K, 6.2 g/kg; P, 1.2 g/kg; Cl, 0.1 g/kg; Na, 0.7 g/kg; and the pH was 8.0 (Egamberdieva et al., 2010).

Three chickpea plants were randomly chosen from the field site and the whole plants including the root system were wrapped in plastic bags, brought to the laboratory and stored at 4°C until further processing. The root system of the collected plants was separated from the shoots and carefully washed under running tap water taking precaution to minimize root injury. Healthy, non-ruptured nodules were carefully collected from root and washed under running water. The nodules were surface sterilized by dipping in 95% ethanol for 1 min and then in 1% NaClO solution for 3 min, and rinsed in sterile distilled water. Sterilized nodules were weighed aseptically (1 g) and macerated in a mortar utilizing phosphate buffered saline (PBS) (20 mM sodium phosphate, 150 mM NaCl, pH 7.4). The nodule juice (1 ml) was placed in a tube containing 9 ml sterile PBS and shaken with a vortex for 1 min. The supernatant was collected, then serially diluted (10^1^–10^5^) and 100 μl aliquots from the appropriate dilutions were spread on Tryptic Soy Agar (TSA), nutrient agar (BD, Difco Laboratories, Detroit, USA), and King's B agar medium (King et al., 1954) in triplicate. The plates were incubated at 28°C and a representative number of colonies that displayed different colony morphologies were picked up from the plates and were re-streaked for the purification of the isolates. In total, 40 bacterial pure cultures were preserved on plates at −80°C.

### Colonization assay and re-isolation of endophytic isolates

The chickpea seeds (*C. arietinum* L., cv Uzbekistan-32) were obtained from the International Center for Agricultural Research in the Dry Areas (ICARDA). Seeds were surface-sterilized for 5 min with 1% NaClO solution followed by 95% ethanol for 3 min, rinsed five times with sterile distilled water and germinated on 1% water agar in the dark at 28°C. The sterility of seeds was tested on TSA agar in incubation plates for 3 days at 28°C. No contaminants were found, indicating that the surface sterilization procedure was effective.

Forty bacterial isolates from the chickpea nodules were grown overnight in TSB broth, 1 ml of each culture was centrifuged, and cell pellets were suspended with PBS (cell density of 10^7^ CFU/ml). Germinated seeds were dipped in bacterial suspension for 10 min, and were aseptically planted in a sterile sand column in the gnotobiotic system glass tubes (30 mm in diameter, 200 mm in length) as described by Simons et al. (1996). The tubes containing 100 g of sterilized washed sand were soaked with 10 ml of Hoagland's plant nutrient solution (Lynch et al., 1990), supplemented with 100 mM NaCl. The seedlings were grown in a growth cabinet with a 16-h light period at 22°C and an 8-h dark period at 16°C, one seed per tube with 10 replicates for each bacterial inoculant for 10 days. To re-isolate the bacteria from the root, the complete sand column was carefully removed from the tube and roots were surface-sterilized using 70% ethanol and 1% NaClO solution and were rinsed five times with sterile water to remove disinfectant. The root samples were macerated with a mortar and pestle, and macerated tissue extracts were serially diluted in PBS and 0.1 ml aliquots were spread on TSA plates and incubated at 28°C for 3–5 days to isolate bacterial endophytes. Of the inoculated isolates, 10 were re-isolated from the plants as endophytes and were characterized for plant growth promoting (PGP) traits.

### Characterization of bacterial isolates

To test whether bacterial isolates were capable of stimulating plant growth, the seeds were surface-sterilized, and inoculated with the bacterial isolates as described above. Plants were grown in glass jars (1,000 ml). The jars were filled with a sterilized mixture of washed sand and vermiculite (1:1), and finally with Hoagland plant nutrient solution (Lynch et al., 1990) supplemented with 100 mM NaCl. The 10 seedlings per treatment in three replication were grown in a plant growth chamber with a 11-h light period at 24°C and an 8-h dark period at 18°C. After 3 weeks, the seedlings were removed from the sand and root and shoot dry weight was determined.

The determination of IAA (indole 3-acetic acid) was assayed as described by Bano and Musarrat (2003). The IAA concentration in culture was calculated using the calibration curve of pure IAA as a standard. The phosphate-solubilizing activity of the bacterial isolates was determined using Pikovskaya agar (Pikovskaya, 1948) containing precipitated tricalcium phosphate. The presence of clearing zones around the bacterial colonies was considered an indicator for positive solubilization activity. The production of chitinase enzymes was performed using colloidal chitin medium and protease activity was encouraged using sterile skim milk agar plates as described in Frändberg and Schnürer (1998), Dutta et al. (2015). The cellulose-degrading ability of the bacterial isolates was analyzed by streaking inocula on cellulose (Sigma-Aldrich, St. Louis, MO) Congo-Red agar media as described by Pratima et al. (2012). Lipase activity of the bacterial isolates was determined using the Tween lipase indicator assay (Howe and Ward, 1976). Furthermore, β-1,3 and β-1,4 glucanase activity was tested using the substrate lichenan (Sigma-Aldrich, St. Louis, MO) in top agar plates (Walsh et al., 1995). The production of HCN by bacterial isolates was determined following Castric (1975). The production of siderophores by the bacterial isolates was determined using chrome azurol S (CAS) agar media as described by Alexander and Zuberer (1991). The antagonistic abilities of selected isolates against pathogenic fungi *F. oxysporum, F. solani, Fusarium culmorum, Alternaria alternata*, and *Botrytis cinerea* were evaluated as described by Egamberdieva et al. (2016c). Briefly, fungal strains were grown in PDA plates for 5 days and small disks of agar piece with fungus were cut and replaced in the middle of fresh PDA plates. Holes (5 mm diameter) were made into PDA plates 2 cm away from fungal disc and 100 μl of bacterial cultures pregrown in TSB broth for 3 days were dropped into a hole. The plates were sealed with Parafilm® M and incubated at 28°C in darkness for 6 days. The growth inhibition zone of fungi was recorded.

### Identification of selected PGPR isolates

The isolation of DNA was carried out according to Töpper et al. (2010). A lysozyme solution [250 μl; 1 mg ml-1TE buffer (10:1, pH 7.4)] was used to re-suspend the filters and was added to a lysis buffer (250 μl; 20 μg proteinase K ml^−1^ 0.5% SDS). After incubation for 30 min at 55°C, 80 μl of 5 M NaCl, and 100 μl of preheated (55°C) CTAB (10% (w/v) hexadecyltrimethylammonium bromide in 0.7% NaCl) were added to the solution. After 10 min incubation at 65°C, 500 μl of chloroform: isoamyl alcohol (24:1) was added to the solution and centrifuged at 16,000 × g for 5 min. A TE buffer was used to re-suspend the DNA, which was precipitated with isopropanol. The 16S rDNA was amplified with polymerase chain reaction (PCR) using the universal forward 16SF: 5′-GAGTTTGATCCTGGCTCAG-3′ and reverse 16SR: 5′-GAAAGGAGGTGATCCAGCC-3′ primers (Yuwa-Amornpitak, 2012).

The PCR products were obtained following the procedure described in Hashem et al. (2016) and verified via gel electrophoresis on a 1.5% agarose gel stained with TAE (Tris-acetate-EDTA). Gels were visualized and digitized using the Fujifilm Imaging System. The PCR product was purified, and nucleotide sequences were determined using the automatic LI-COR DNA Sequencer 4000 L (Lincoln, USA). The nucleotide sequences were determined using a sequenator. The sequences were identified using the basic local alignment search tool (BLAST) and comparisons with the GenBank nucleotide data bank from the National Center for Biotechnology Information (NCBI) (http://www.ncbi.nlm.nih.gov/). Molecular Evolutionary Genetics Analysis (MEGA) software version 6.0 was used to create the phylogenetic tree of the selected bacterial isolates. The 16S rRNA gene sequences of the new isolates were registered in GenBank (NCBI) with the following accession numbers: *Bacillus cereus* isolate NUU1—KU975367.1, *Achromobacter xylosoxidans* isolate NUU2—KU975368.1, *Bacillus thuringiensis* isolate NUU3—KU975369.1, *Bacillus subtilis* isolate NUU4—KU975370.1. MEGAsoftware version 6.0 was used to create the phylogenetic tree of the selected bacterial isolates.

### Plant growth, symbiotic performance under salinated soil

Pot experiments were conducted to investigate the effect of endophytes on symbiotic performance of rhizobia with host and plant growth. The soil used for the pot experiments was described above. The treatments were as follows: (i) seeds inoculated with *Mesorhizobium ciceri* strain IC53 alone and (ii) *M. ciceri* IC53 combined with endophytic bacterial isolates. Bacterial inoculants were prepared and the germinated seeds were inoculated by immersing seeds in the cell suspensions as described above. *M. ciceri* IC53 was grown in yeast extract-mannitol (YM) (Difco) and endophytic bacteri in TSB medium. For co-inoculation, the cell suspension (cell density of 10^7^ CFU/ml) of two bacterial isolates was mixed in a 1:1 ratio and vortexed vigorously to yield a homogenous suspension. One seed was sown per plastic pot, each pot containing 500 g of saline soil, at a depth of ~1.5 cm. Each treatment contained six plants with three replications. The plants were grown in a shaded greenhouse for 30 days. The temperature ranged between 28 and 32°C during the day and between 18 and 22°C at night. At harvest, the shoot and root lengths and dry weight and nodulation were determined.

### Isolation of pathogenic fungi

A chickpea field located in Syrdarya province, semi arid region of Uzbekistan was chosen to sample diseased plants. The field is characterized by high salinity (EC 7.5 dS/m), and a high share of plants showing stem root disease were found. The plants with external symptoms such as leaf browning, brown stems, and rooted taproot were collected and carried to the laboratory. The roots that showed disease symptoms were cut into 0.5 cm pieces, and were surface disinfected by dipping into 70% ethanol, then 1% NaOCl for 5 min. Thereafter, roots were washed with sterile distilled water three times and dried on sterile blotting paper. Sterile root pieces were placed on the surface of potato dextrose agar (PDA) (BD, Difco Laboratories, Detroit, USA) supplemented with chloramphenicol (Oxoid, UK) (150 ppm) and incubated at 25°C in the dark. After 8–10 days, three fungal colonies which differed morphologically were isolated, subcultured and purified. Morphological characteristics such as colony appearance were determined from fungal isolates grown on PDA medium after 5 days at 28°C. The formation of conidia, chlamidospores was examined microscopically using light microscope (Olympus BX50).

### Plant pathogenicity test

For plant pathogenicity tests, three purified fungal isolates taken from diseased roots were grown on PDA medium at 25°C for 6 days. Sterile saline containing 0.1% (v/v) Tween 20 was poured onto plate cultures, gently washed with a sterile glass spreader and a suspension was collected in a sterile tube. The fungal suspension was filtered through glass wool to remove hyphal fragments and the suspension was centrifuged and resuspended with distilled sterilized water. The concentration of spores was counted using a hemocytometer and diluted to 10^8^ spores ml^−1^. For the pathogenicity assay, chickpea seeds were surface-sterilized with 1% NaClO solution followed by 95% ethanol for 5 min, then rinsed several times with sterile distilled water. The sterile seeds were germinated on 1% water agar in the dark at 28°C. The seedlings were dipped in a suspension of spores for 5 min, and then sown in sterile potting soil. Four treatments were used, seeds inoculated with three fungal isolates, and seeds without any treatment. Each treatment contained three replicated blocks (each block has 24 plants), making up a total of 72 plants for each treatment. Plants were grown under greenhouse conditions and were examined for root rot symptoms after 30 days. Among three isolates only one isolate showed root rot symptoms and was collected to re-isolate the fungus from root tissue as described above. Among three fungal isolates only one isolate caused brown, discolored taproots in chickpea. The isolation and purification of fungal isolates from affected tissues was performed as described above.

### Identification of fungal isolates

The fungal isolates were grown on PDA agar plates at 25°C for 7 days. The fungal mycelium was collected from the surface of the agar with a sterile spatula and transferred to a sterile tube. The mycelium was washed in sterile tap water, centrifuged for 10 min at 4,000 × g, and then freeze-dried at −20°C. DNA was extracted by using the FastDNA Spin Kit (Qbiogene, CA, USA), following the manufacturer' instructions. PCR amplification of the rDNA ITS region was performed using fungal primer pairs ITS1 and ITS4 according to Abd-Elsalam et al. (2003). The PCR product was purified, and nucleotide sequences were determined using a DNA sequencer (4000 L, Lincoln, USA). The sequences of the fragments were identified using the basic local alignment search tool (BLAST) (http://www.ncbi.nlm.nih.gov/) with accession number KR528471.1 (*F. solani*).

### Biological control of root rot

Four endophytic bacterial isolates were tested for their ability to control root rot in chickpea caused by *F. solani* under greenhouse conditions. For the soil infestation, *F. solani* was grown in Chapek-Dox medium at 28°C for 4 days and the suspension was filtrated with sterile glass wool to remove the mycelium. The concentration of spores in the suspension was adjusted to 10^7^ spores ml^−1^ and mixed thoroughly with the potting soil to obtain ~10^7^ spores kg^−1^ soil. The sterile seedlings were dipped in bacterial suspension of 1 × 10^8^ CFU ml^−1^. The inoculated seedlings were sown in plastic pots filled with natural saline soil infested with *F. solani* and each treatment contained four groups of 24 plants. The plants were grown under greenhouse conditions at a temperature range of 28–32°C day and 20–22 night. The plants were grown for 30 days and removed at harvest from soil, washed and examined for root rot symptoms as indicated by browning and lesions. In healthy plants no disease symptoms were detected.

### Field experiment

#### Plant growth, nutrient uptake, and yield

The experimental design of the field trials in the salinated area of the Syrdarya province was initiated as described by Egamberdieva et al. (2014). The mean temperature of the growing season in 2014 was 17–19°C (April to May) and 36–38°C (June to July). The experimental plots (10 m^2^) were arranged in a randomized block design with six replicates per treatment. The treatments were as follows: (i) un-inoculated control, (ii) seeds inoculated with *M. ciceri* strain IC53, and (iii) a combined inoculation of seeds with *M. ciceri* IC53 and *B. subtilis* NUU4. The bacterial inoculants were prepared as described above. *M. ciceri* was grown overnight in TY broth and the endophytic isolates were grown in TSB broth. For co-inoculation, cell suspensions of both strains (10^7^ CFU/ml) were mixed in a 1:1 ratio. Chickpea (variety Uzbekistan-32) seeds were planted by hand in each plot in the beginning of April and irrigated by furrow irrigation. Six plants from each treatment were harvested after 2 months and plant shoots were separated from roots and dried to a constant weight at 100°C. The shoots were separated from the roots and dried in an oven at 75°C for 48 h and then powdered. Total nitrogen (Nt) was determined after dry combustion using a CNS elemental analyzer (LECO Corporation, St. Joseph, MI) according to DIN ISO 15178 (2001). The P, K, and Mg contents were analyzed according to DIN ISO 38414-S (1983). The number of pods and nodules per plant root were determined. Seed yields, taken from the two central rows of each plot (m^2^ per plot), were estimated at maturity (3 months after sowing).

#### Plant physiological properties

To determine the chlorophyll content, leaf samples (0.5 g) were extracted in acetone (80%) and centrifuged at 10,000 × g for 10 min. The absorbance of the supernatant was recorded at 645 and 663 nm (T80 UV/VIS Spectrometer, PG Instruments Ltd, USA) against the solvent (acetone) (Arnon, 1949). To determine the soluble leaf proteins, the fresh leaves of each plant (*N* = 5) were frozen in liquid nitrogen, ground using a cold mortar, and macerated in 1.0 ml of 100 mM Tris buffer (pH 8.0). The extract was then centrifuged at 27,000 × g for 10 min at 4°C. The soluble leaf protein content was measured according to Bradford (1976).

Hydrogen peroxide (H_2_O_2_) content in chickpea leaves was evaluated as described by Mukherjee and Choudhuri (1983). The acetone and was used to extract leaf samples, and the supernatant (200 μl) was mixed with 0.04 ml of 0.1% TiO_2_ and 0.2 ml NH_4_OH (20%). The solution was mixed with 0.8 ml H_2_SO_4_, centrifuged for 15 min at 6,000 × g and the supernatant was read at 415 nm. The method described in Bates et al. (1973) was used for the estimation of the proline content in chickpea leaves. Briefly, leaf samples (0.5 g) m were extracted in sulfosalicylic acid (3%) and centrifuged for 30 min at 3,000 × g. The acid ninhydrin solution contains 1.25 g ninhydrin, 30 ml glacial acetic acid and 20 ml of 6 M phosphoric acid. The supernatant (2.0 ml) was mixed with the acid ninhydrin solution and glacial acetic acid and incubated for 10 min at 100°C. The reaction was stopped by placing the tubes in an ice container, and proline was separated with 4 ml toluene and finally, optical density was measured at 520 nm.

### Statistical analyses

Data obtained from the plant morphological and biochemical studies, as well as the number of diseased plants were subjected to analysis of variance (ANOVA) with SPSS software (version 15). The results are presented as average means and standard error (SE). The difference between means was compared by a high-range statistical domain (HSD) using Tukey's test. The treatment means were separated by the least significant difference (LSD) test at *P* < 0.05.

## Results

### Isolation, selection, and characterization of plant growth promoting endophytic bacteria

A total of 40 bacterial isolates were isolated from the surface-sterilized nodules of chickpea grown in saline soil from the Syrdarya province of Uzbekistan. The capability of 40 isolated endophytic bacteria to colonize and persist in plant hosts was tested by studies in a gnotobiotic sand system. Of these forty, 10 isolates colonized the plants at levels ranging from 3.01 to 5.4 log_10_ CFU/g (fresh weight) (Table 1). The endophytes EB2, EB6 EB9, and EB10 were able to colonize root tissue with higher densities than other isolates at titers between 4.60 and 5.45 log_10_ CFU/g (fresh weight).

**Table 1:** Characterization of endophytic bacterial isolates.

**Bacterial strains**	**Colonization (log10 CFU/g (root fw))^a^**	**Plant growth^b^**					**Exo-enzymes^c^**					**Antagonistic activity^c^**				
		**Shoot**	**Root**	**HCN^b^**	**PSB^b^**	**Siderophore^b^**	**IAA (μg/ml)^b^**	**Lipase**	**Protease**	**Cellulase**	**Chitinase**	***F. oxysporum***	***F. solani***	***F. culmorum***	***A. alternata***	***B. cinerea***
EB1	3.01 ^b^^c^	0.042 ^a^^b^^c^	0.016 ^b^	–	–	–	–	–	–	–	+	–	–	–	–	–
EB2	4.60 ^a^^b^^c^	0.054 ^a^	0.018 ^a^^b^	–	–	–	6.2	+	+	+	+	+	+	+	–	–
EB3	4.43 ^a^^b^^c^	0.036 ^c^	0.013 ^c^	–	+	–	–	–	+	–	–	–	–	–	–	–
EB4	3.46 ^b^	0.038 ^a^^b^^c^	0.014 ^b^^c^	+	–	–	3.8	+	–	–	–	–	–	–	–	–
EB5	2.51 ^c^	0.041 ^a^^b^	0.015 ^a^^b^^c^	–	–	–	–	–	–	+	+	+	+	+	–	–
EB6	5.45 ^a^	0.038 ^a^^b^^c^	0.018 ^a^^b^	+	+	+	4.0	–	–	–	–	–	–	–	–	–
EB7	3.02 ^b^^c^	0.037 ^b^^c^	0.015 ^a^^b^^c^	–	–	–	5.1	–	+	+	+	+	+	+	–	–
EB8	2.89 ^b^^c^	0.037 ^b^^c^	0.015 ^a^^b^^c^	–	+	–	2.9	+	+	–	–	–	–	–	–	–
EB9	4.92 ^a^^b^	0.046 ^a^^b^	0.014 ^b^^c^	–	–	–	–	–	+	–	+	–	+	+	–	–
EB10	5.01 ^a^^b^	0.053 ^a^^b^	0.019 ^a^	+	+	+	8.6	+	+	+	+	+	+	+	+	+

a *Plants were grown under gnotobiotic system for 10 days*.

b *Untreated plants with bacteria (control): shoot—0.37 and root—0.013 g/plant*.

c *All tests were conducted with the addition of 2% NaCl*.

“+” *positive*. “–“ *negative*.

*Different letters in root colonization and plant growth data indicate significant differences based on Turkey's HSD test at P < 0.05*.

Ten bacterial isolates were also screened for multiple plant growth promoting traits and for their plant growth promoting attributes under salt stress. Only four isolates EB2, EB6, EB9, and EB10 significantly stimulated root dry biomass of chickpea by 41, 38, 5, and 46%, whereas shoot dry biomass increased by 45, 5, 24, and 43%, respectively Other isolates did not show any significant impact on plant growth (Table 1).

**Figure 1:**
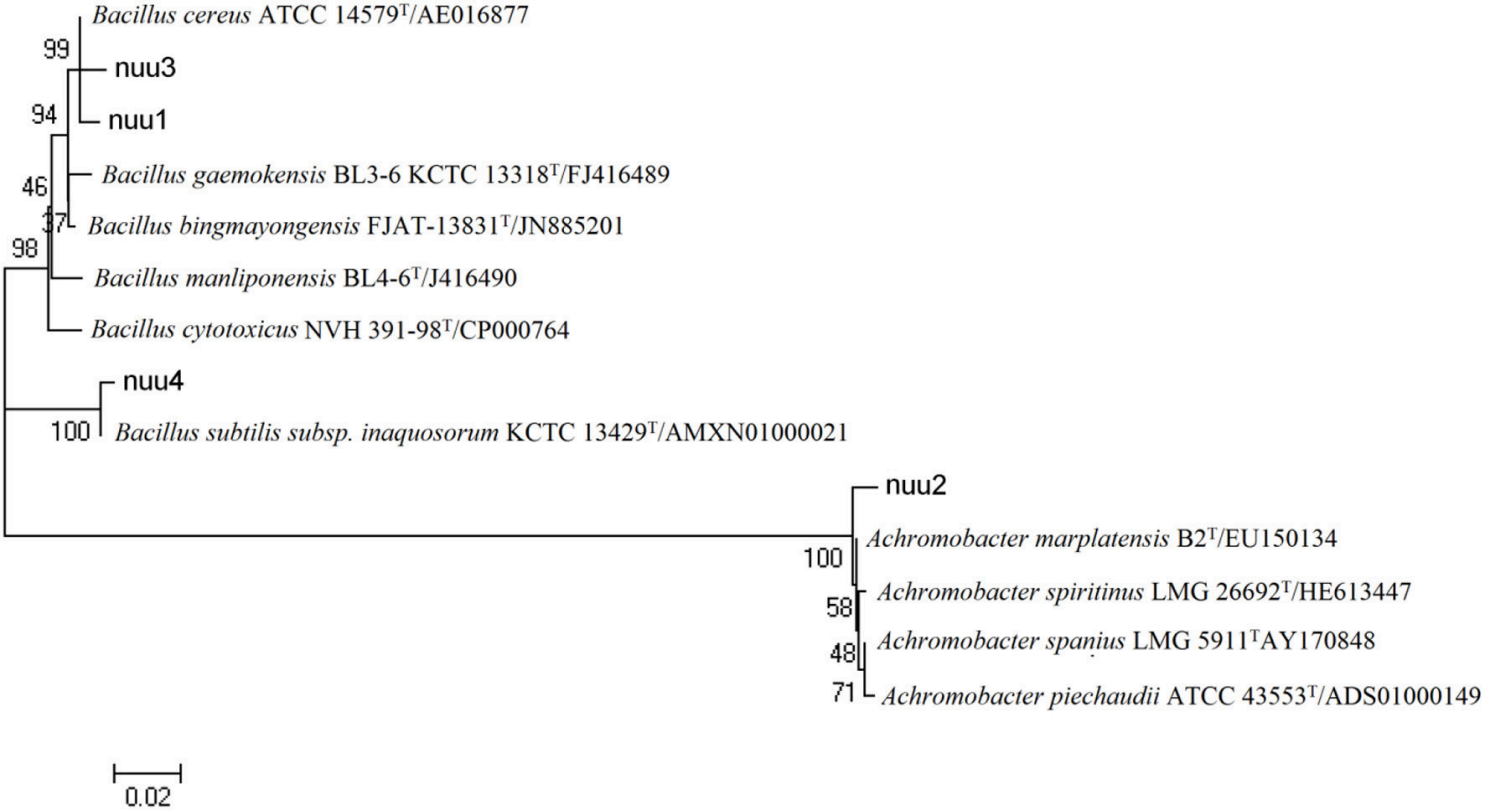
Phylogenetic tree based on alignment of nucleotide sequences of the 16S rRNA genes among selected bacterial isolates isolated from chickpea root and related genera. The bacterial isolates are indicated as follows (*Bacillus cereus* strain, nuu1; *Achromobacter xylosoxidans* strain, nuu2; *Bacillus thuringiensis* strain, nuu3; *Bacillus subtilis* strain, nuu4).

Most of the bacterial isolates exhibited one or more plant growth-promoting activities (Table 1). The highest amount of IAA production was observed with EB10 isolate (8.6 μg ml^−1^) and EB2 isolate (6.2 μg ml^−1^). The isolates EB1, EB3, EB5, and EB9 did not show any IAA production. Six isolates, except EB1, EB3, EB4, and EB6, were able to produce one or more cell wall degrading enzymes. The isolates EB2 and EB10 *B* produced lipase, protease, cellulase, and chitinase emzymes. Antagonistic activity was recorded for endophytes against plant pathogenic fungi such as *F. oxysporum, F. solani, F. culmorum, A. alternata*, and *B. cinerea*. The isolate EB2 was highly effective against *Fusarium* pathogens, and only isolate EB10 showed antagonistic activity against all of the fungal pathogens (Table 1). HCN and siderophores were produced by two isolates, EB6 and EB10, and both were able to solubilize phosphate. Four selected bacterial isolates which showed best plant stimulating performance and beneficial traits were identified and chosen for further studies. Based on the nucleotide identity and phylogenetic analysis of the 16S rRNA gene sequences, EB2 (NUU1) was found to be 99% similar to *B. cereus* ATCC 14579 (NC_004722.1.), EB6 (NUU2) was *A. xylosoxidans* A8 (NC_014640.19, EB9 (NUU3) was *B. thuringiensis* serovar konkukian str. 97-27 (NC_005957.1), and EB10 (NUU4) was *B. subtilis* subsp. *subtilis* str. 168 (NC_000964.3). Figure 1 shows the phylogenetic tree of selected isolates (NUU1, NUU2, NUU3, and NUU4) created by using their 16S rRNA sequences.

### Improvement of plant growth and chickpea-rhizobia symbiotic performance

**Figure 2:**
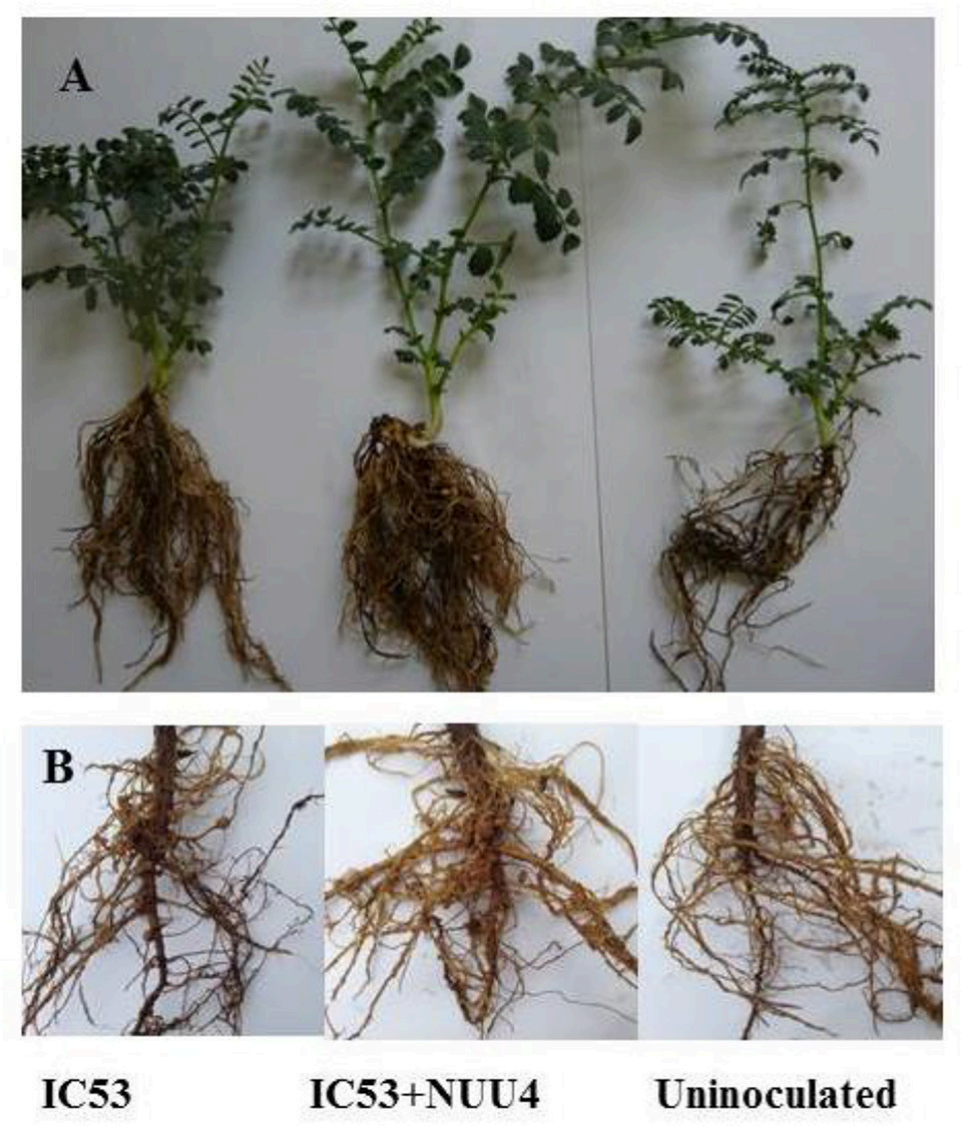
Growth of chickpea plants after inoculation with *Mesorhizobium ciceri* IC53 alone or with the combination of *Mesorhizobium ciceri* IC53 and *Bacillus subtilis* NUU4 in pots **(A)** and under field condition **(B)**.

The four selected isolates (*B. cereus* NUU1, *A. xylosoxidans* NUU2, *B. thuringiensis*, NUU3, and *B. subtilis* NUU4) were evaluated for their ability to improve plant growth and chickpea-rhizobia symbiotic performance in pots under saline soil conditions. A single-strain inoculation with the *M. ciceri* IC53 strain significantly improved the shoot height and nodule number compared to the un-inoculated plants. The shoot height increased 17% and the nodule number, on average, increased two-fold (Table 2). In comparison with un-inoculated chickpea, the endophytic isolates NUU1, NUU2, NUU3, and NUU4 increased the root dry weights by 24, 30, 30, 40% and shoot dry weights by 11, 13, 18, and 20% respectively. In comparison with the single-strain inoculation (*M. ciceri*), the co-inoculation of a *Mesorhizobium* symbiont with the endophytic isolate NUU4 further increased shoot and root weights and nodule number (Table 2, Figure 2A). The shoot height increased by 14%, the root and shoot dry weights increased 20 and 24% compared to single inoculated plants with *M. ciceri*, respectively. The co-inoculation of *M. ciceri* IC53 with *B. subtilis* significantly improved the nodulation of chickpea more than two-fold (Table 2).

**Table 2:** The effect of endophytic bacteria alone and in combination with *Mesorhizobium ciceri* on chickpea shoot height (SH), nodule number (NN), root dry weight (RDW), and shoot dry weight (SDW) under saline soil conditions.

**Treatments**	**SH (cm/plant)**	**NN (per/plant)**	**RDW (g/plant)**	**SDW (g/plant)**
Control	13.7 cd±1.48	2.0 f±0.81	0.100 d±0.018	0.175 g±0.013
*M. ciceri* IC53	16.1 b±0.75	6.0 cd±0.82	0.120 cd±0.011	0.185 fg±0.013
*B. cereus* NUU1	13.3 d±1.09	2.7 ef±0.95	0.124 bc±0.007	0.195 dfg±0.011
*B. cereus* NUU1 + *M. ciceri* IC53	15.3 bc±1.10	6.0 cd±1.82	0.122 bcd±0.015	0.190 dfg±0.008
*A. xylosoxidans* NUU2	13.7 cd±1.23	3.7 def±0.95	0.130 bc±0.008	0.198 cdf±0.010
*A. xylosoxidans* NUU2 + *M. ciceri* IC53	16.6 b±1.65	9.0 b±1.90	0.135 abc±0.024	0.218 b±0.010
*B. thuringiensis* NUU3	13.7 d±1.47	3.8 cde±0.95	0.130 bc±0.013	0.208 bcd±0.017
*B. thuringiensis* NUU3 + *M. ciceri* IC53	16.6 b±1.04	9.0 bc±1.50	0.135 ab±0.025	0.215 bc±0.013
*B. subtilis* NUU4	14.2 cd±0.68	6.0 cd±1.82	0.140 abc±0.008	0.210 bcd±0.008
*B. subtilis* NUU4 + *M. ciceri* IC53	18.5 a±0.31	14.5 a±2.01	0.145 a±0.012	0.230 a±0.022

*The plants were grown at a temperature range of 28–32°C in greenhouse condition for 30 days; each treatment contained six plants with three replications; different letters indicate significant differences based on Turkey's HSD test at P < 0.05*.

### Biological control of chickpea root rot under saline soil

The endophytic bacterial isolates were screened for the biocontrol of chickpea root rot under greenhouse conditions. The fungal isolate from chickpea roots with disease symptoms was found to be a causative agent of root rot after plant pathogenicity tests. The analysis of DNA sequences showed that the isolate revealed 99% sequence identity to *F. solani* strain QK1409120101 (GenBank accession number KR528471.1) and was therefore assigned to *F. solani*. The isolate was used in biological control assays to estimate biocontrol capability of endophytic isolates of chickpea against root rot under saline soil conditions.

The four selected isolates *B. cereus* NUU1, *A. xylosoxidans* NUU2, *B. thuringiensis* NUU3, and *B. subtilis* NUU4 were chosen to evaluate their ability to suppress chickpea root rot caused by *F. solani*. In non-infested soil, the portion of diseased plants was 6%, while in the presence of the pathogen, the portion of plants that displayed disease symptoms increased to 27%. The isolate *B. cereus* NUU1 demonstrated a disease reduction up to 17% compared to *Fusarium*-infected control plants that showed 27%. The isolates *A. xylosoxidans* NUU2 and *B. thuringiensis* NUU3 were not able to protect chickpea root against the fungal pathogen. *B. subtilis* NUU4 demonstrated the best performance reducing diseased plants by 8%, whereas Fusarium-infected control plants showed 27% diseased plants (Figure S1).

### Plant response to bacterial inoculants under field conditions

#### Plant growth and yield

**Figure 3:**
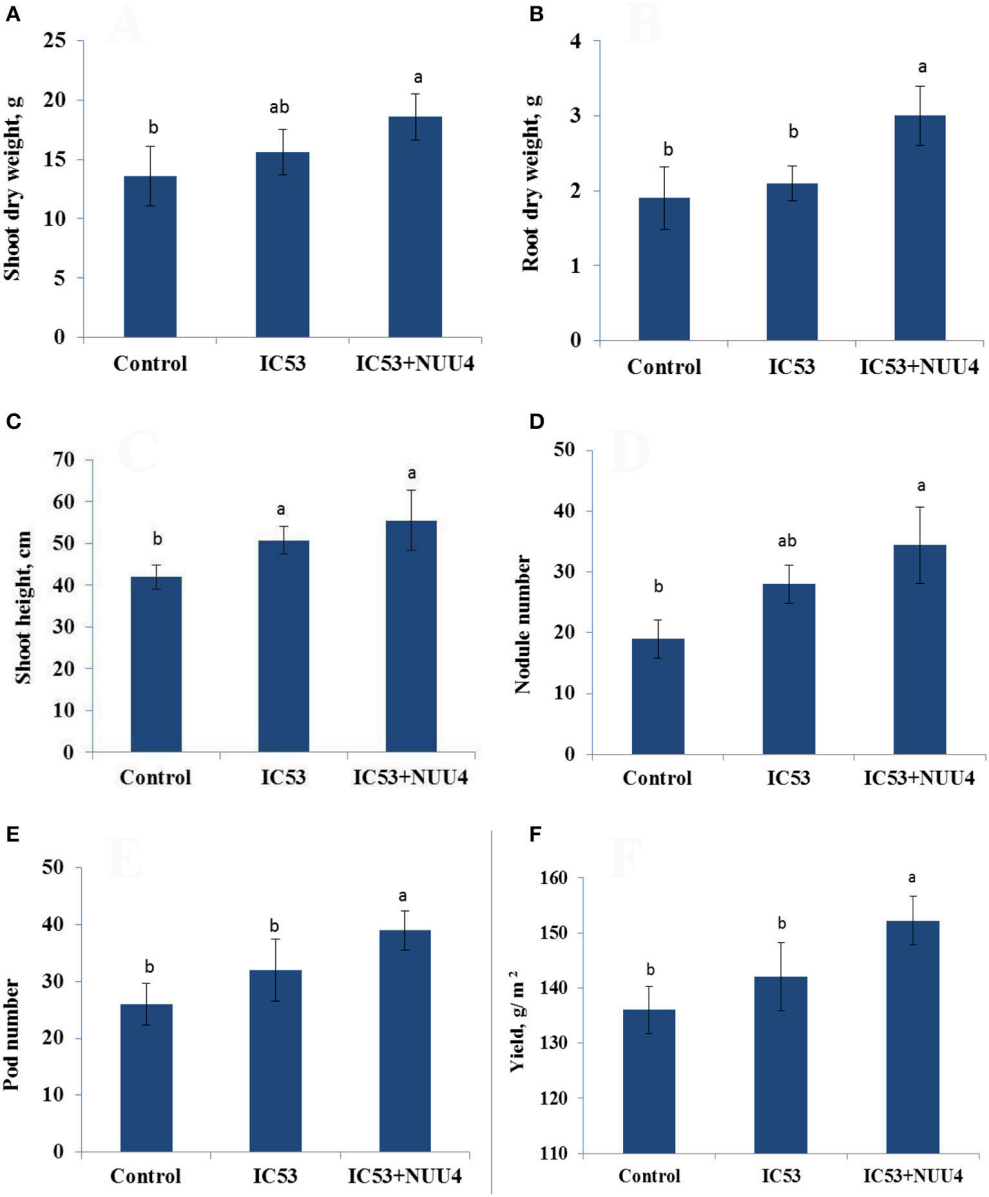
Effects of seed inoculation with the combination of *Mesorhizobium ciceri* IC53 and *Bacillus subtilis* NUU4 and with *Mesorhizobium ciceri* IC53 strain alone on shoot **(A)** and root **(B)** dry weight, shoot **(C)** height, nodule number **(D)**, pod number **(E)**, and seed yield **(F)** grown under saline soil conditions. Columns represent the means of six plants (*N* = 6) and error bars show the standard error. Column means marked by different letters indicate significant differences based on Turkey's HSD test at *P* < 0.05.

The isolate *B. subtilis* NUU4, which demonstrated a positive effect on chickpea growth, symbiotic performance, and biological control of root rot in the preliminary pot experiments, was tested for efficiency under field conditions. The results indicate that a combined inoculation of *B. subtilis* NUU4 and *M. ciceri* IC53 was effective in terms of chickpea growth promotion, stress tolerance, nodulation, pod formation, and yield compared to the un-inoculated control and the treatment of IC53 alone. In the case of the single inoculation with IC53, the shoot height, nodule number, pod number, and yield were significantly (*p* < 0.05) increased, and there was no significant effect on root and shoot dry weights, (Figures 3A–D). The root dry weight of chickpea significantly increased (*P* < 0.05) by dual inoculation compared to control and single inoculation by 87 and 35% respectively (Figure 3B). For the dual inoculated chickpea seeds with IC53 and NUU4, plants contained 81 and 22% more nodules compared to the un-inoculated control and the single inoculation of IC53, respectively (Figures 2B, 3D). The pod number and yield of inoculated chickpea with a combined inoculation were 39 and 13% higher compared to the un-inoculated plants, respectively, and 12 and 7% with the single inoculation of IC53 (Figures 3E,F).

#### Nutrient acquisition

**Figure 4:**
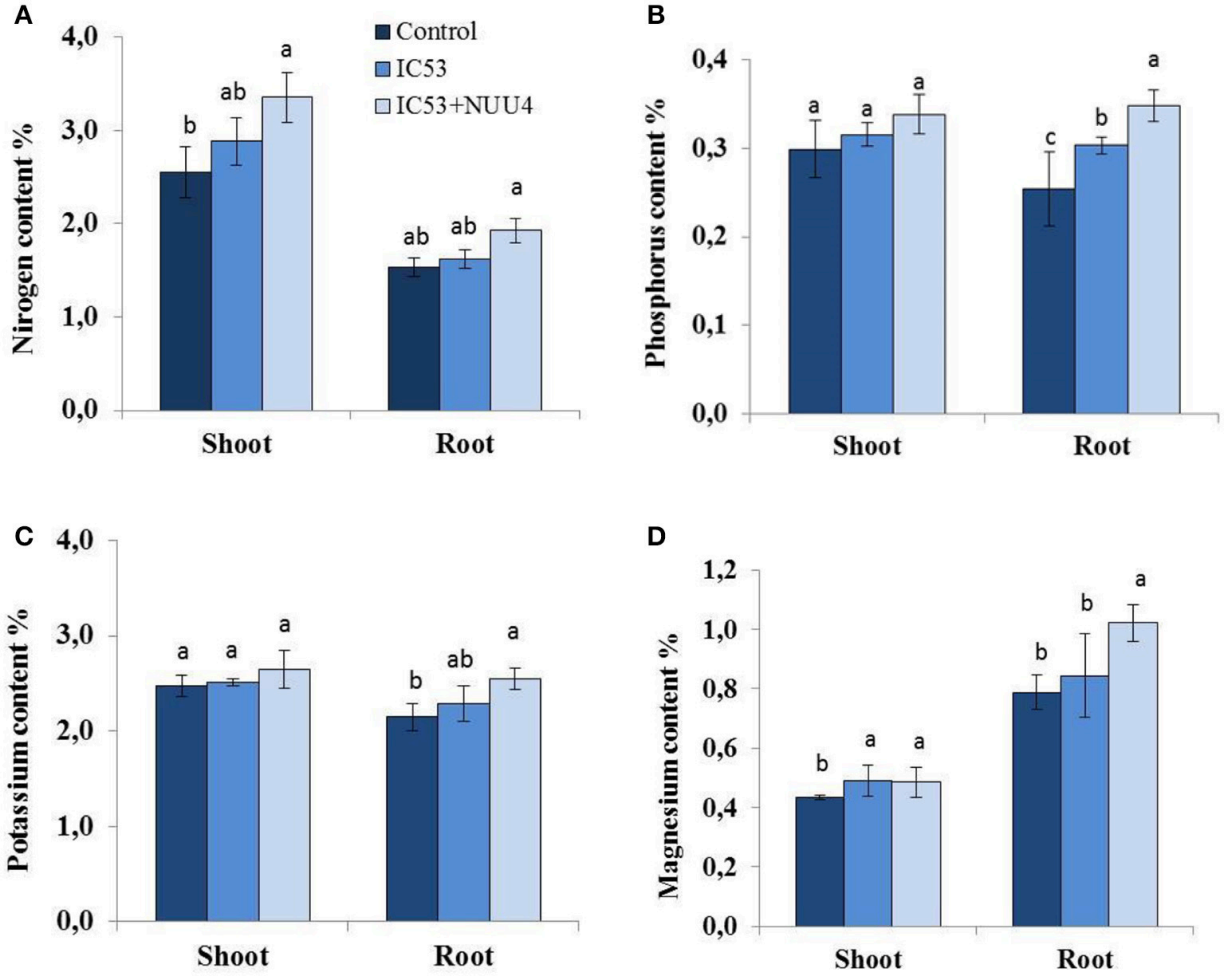
Effects of seed inoculation with the combination of *Mesorhizobium ciceri* IC53 and *Bacillus subtilis* NUU4 and with *Mesorhizobium ciceri* IC53 strain alone on chickpea shoot and root **(A)** nitrogen, **(B)** phosphorus, **(C)** potassium, and **(D)** magnesium contents grown under saline soil conditions. Columns represent the means of six plants (*N* = 6) and error bars show the standard error. Column means marked by different letters indicate significant differences based on Turkey's HSD test at *P* < 0.05.

Under saline conditions, nitrogen (N), phosphorus (P), potassium (K), and magnesium (Mg) contents of roots and shoots in the un-inoculated plants were lower compared to single and dual inoculated chickpea with IC53 and NUU4 (Figures 4A–D). The nutrient content responded positively to both inoculation treatments, and the chickpea inoculated with the IC53 strain and IC53 combined with NUU4 contained 12 and 31 and 5 and 26% more nitrogen in the shoots and roots compared to the un-inoculated plants, respectively (Figure 4A). The phosphorus content in the shoots of plants inoculated with both IC53 and IC53 combined with NUU4 showed no significant difference compared to the un-inoculated control (Figure 4B). However, the highest phosphorus content was detected from the root tissues inoculated with IC53 combined with NUU4 (36%) and with IC53 only (19%), compared to the un-inoculated control plants. A slightly similar phenomenon was detected for potassium content when chickpea was inoculated with IC53 combined with NUU4. The K content increased by 18% in the roots of chickpea treated with the dual inoculation of the microbes (Figure 4C). Magnesium content of chickpea shoots was not affected by both treatments, whereas plant roots contained significantly more (up to 29%) Mg after inoculation with IC53 and NUU4 compared to the un-inoculated plant tissues (Figure 4D).

#### Physiological parameters

The chlorophyll content in chickpea leaves was lower in plants without the bacterial treatments. The plants inoculated with the combination of *M. ciceri* IC53 and *B. subtilis* NUU4 showed 51 and 26% higher photosynthetic pigments compared to the un-inoculated plants and the single inoculation with *M. ciceri* IC53, respectively (Table 3). The soluble leaf protein in chickpea also responded positively to both microbial inoculations. The combination of the two bacteria produced better results since co-inoculated chickpea tissues contained more leaf proteins (26%) than the un-inoculated tissues (Table 3).

**Table 3:** Effects of seed inoculation with *Mesorhizobium ciceri* IC53 alone and *Mesorhizobium ciceri* IC53 combined with *Bacillus subtilis* NUU4 on the contents of chlorophyll, protein, hydrogen peroxide, and proline of chickpea grown under saline soil conditions.

**Treatment**	**Chlorophyll**	**Protein**	**Hydrogen peroxide**	**Proline**
Control	1.32 ± 0.03 b	1.83 ± 0.02 b	7.87 ± 0.67 a	38.43 ± 3.1 bc
*M. ciceri* IC53	1.58 ± 0.02 ab	2.12 ± 0.01 ab	6.51 ± 0.63 b	42.87 ± 2.3 b
IC53 + *B. subtilis* NUU4	2.00 ± 0.01 a	2.31 ± 0.02 a	5.61 ± 0.87 c	49.79 ± 3.8 a

*The plants were grown under field conditions for 60 days; different letters indicate significant differences based on Turkey's HSD test at P < 0.05. Chlorophyll, mg/g fresh weight; Protein, mg/g fresh weight; hydrogen peroxide, μM/g fresh weight; Proline, nM/g fresh weight*.

Hydrogen peroxide and proline acts as stress-related signaling molecules that involved in the regulation of various abiotic and biotic stresses in plants. The content of hydrogen peroxide (H2O2) in the chickpea leaves was 7.87 (μM/g fresh weight). The single inoculation of plants with *M. ciceri* IC53 decreased H2O2 by 18% and a dual inoculation with *M. ciceri* IC53 and *B. subtilis* NUU4 by 29%. The plants grown under saline soil conditions had lower concentrations of proline (38.43 nM/g fresh weight) compared to the inoculated plants (49.79 nM/g fresh weight). Proline was increasingly produced by plants as a response to both the single and dual microbial inoculations. A combined inoculation of chickpea with *M. ciceri* IC53 and *B. subtilis* NUU4 significantly increased proline contents compared to the un-inoculated plants (29%), whereas a single inoculation with *M. ciceri* IC53 increased proline only by 11%.

## Discussion

We studied plant growth promoting endophytic bacteria isolated from chickpea to evaluate their role in biocontrol of root rot caused by *F. solani*, besides symbiotic performance and stress tolerance of chickpea under saline soil conditions. The endophytic bacteria which showed best plant beneficial properties were identified as *B. cereus, A. xylosoxidans, B. thuringiensis*, and *B. subtilis*. The species *A. xylosoxidans* was never previously observed as an endophytic bacterium associated with chickpea. This study reports the first detection of *A. xylosoxidans* in chickpea nodule tissue. Interestingly, some studies reported that *A. xylosoxidans* was able to form nodules in Mesquite (*Prosopis juliflora*) (Benata et al., 2008), cowpea (*Vigna unguiculata*) (Azarias Guimarães et al., 2012), and soybean (*Glycine max*) (Wedhastri et al., 2013). Several endophytic bacteria showed a potential for improving plant growth and stress tolerance (Hashem et al., 2016). These properties were also detected in the case of our endophytic bacterial isolates. The four selected isolates of *B. cereus* NUU1, *A. xylosoxidans* NUU2, *B. thuringiensis* NUU3, and *B. subtilis* NUU4 stimulated the root and shoot growth as well as the nodulation of chickpea. Similar observations were reported by Hashem et al. (2016), stating that *B. subtilis* stimulated root and shoot growth, nodulation and nutrient uptake of *Acacia gerrardii* under salt stress. Furthermore, the best plant growth promoting strain *B. subtilis* NUU4 showed good biocontrol capacity against chickpea root rot caused by *F. solani* under saline soil conditions. Chickpea root rot is a common disease in many countries of the world (Cabral et al., 2016). We isolated pathogenic fungi from infected chickpea roots and identified them as *F. solani*. This is the first report of *F. solani* causing chickpea root rot in a saline soil of Uzbekistan. Chemical plant protection agents are widely used to control various root pathogens, nonetheless they continue to spread and potentially harmful biocides accumulate in soils which are already impacted by other detriments such as salinity. Endophytic microbes are known as potential biocontrol agents of soil borne diseases. The ability of endophytic bacteria colonizing internal plant tissues to protect host plants from soilborne pathogens was recently reviewed by Eljounaidi et al. (2016). For example, the endophytic bacterium *Pseudomonas fluorescens* PICF7 antagonistic against *Verticillum dahliae*, was found to be an effective biological control agent against verticillium wilt of olive (Mercado-Blanco et al., 2004). Several mechanisms behind the plant beneficial effects are reported, including the synthesis of plant growth regulators, antifungal compounds, cell wall degrading enzymes, and/or the modulation of the physio-biochemical processes in plants (Park et al., 2013; Cho et al., 2015; Parray et al., 2015). In our study, the isolate *B. subtilis* NUU4 produced IAA, HCN, siderophores, cell wall degrading enzymes, and demonstrated antagonistic activity against *F. oxysporum, F. solani, F. culmorum, A. alternate*, and *B. cinerea*. Those traits, alone or in combination, may result in an enhanced root growth, nutrient availability to the plants, and a reduction in pathogen infection.

The endophytic bacteria were also effective in chickpea rhizobia symbiotic interactions under saline arid soil conditions. It has been proposed that root associated plant beneficial bacteria living in a free or an endophytic lifestyle may directly or indirectly contribute to the infection and colonization processes of the rhizobium-host association (Egamberdieva et al., 2016b). In the soil-root system, endophytic bacteria do not interfere with the capability of rhizobia to form nodules in the plant roots, even though they may enhance nodulation and plant growth (Egamberdieva et al., 2016b). There are many reports on improved legume-rhizobia symbiotic performance by PGPR e.g., soybean (*G. max* L.) (Egamberdieva et al., 2016a), thal tree (*A. gerrardii*) (Hashem et al., 2016), chickpea (*C. arietinum* L.) (Panjebashi et al., 2012; Yadav and Verma, 2014), and peanut (*Arachis hypogaea*) (Badawi et al., 2011). Accordingly, we observed that the selected salt tolerant PGPR isolates improved the symbiotic performance of *M. ciceri* under saline soil conditions. The nodule number in single inoculated chickpea plants were slightly higher compared to untreated plants, which demonstrate evidence for the impact of salt stress on the symbiotic performance of rhizobia. Salinity above 3 dS m^−1^ was reported to inhibit nodulation in chickpea, except for salt tolerant genotypes which can nodulate salinity up to 6 dS m^−1^ (Rao et al., 2002). When colonizing plant tissues, endophytic bacteria produced various biological active metabolites, which resulted in improved root growth, higher stress tolerance, and the modulation of plant defense mechanisms (Bordiec et al., 2011). The endophytic bacteria have the capability to synthesize cell wall-degrading enzymes, such as cellulase, that are predicted to participate in the penetration of rhizobia into the root cortex and form nodules (Egamberdieva et al., 2013). In another study, Huang et al. (2011) found that the colonization of *Bacillus* and bacteroid formation inside plant cortical cells was similar compared to the infection of root hairs by rhizobia.

The nutrient acquisition in plants under salt stress is generally affected by the antagonistic impact of sodium (Attia et al., 2008) and a reduced root system (Egamberdieva et al., 2016b). We observed an improved N, P, K, and Mg uptake in chickpea inoculated with the combination of *M. ciceri* and *B. subtilis* under saline soil conditions. The stimulated root system induced by endophytic bacteria could explain the enhanced capacity of the plant to acquire and utilize more nutrients. Root associated microbes are also capable of solubilizing mineral nutrients and facilitating their availability to plants, increasing nutrient uptake (Setiawati and Mutmainnah, 2016). For example, phosphate-solubilizing *Pseudomonas* in combination with *Sinorhizobium ciceri* increased P uptake by chickpea (Messele and Pant, 2012). The isolate *B. subtilis* NUU4 was also able to solubilize phosphate, thus providing more phosphorus to chickpea plants.

The inoculation of chickpea with the endophytic isolates also affected several physiological properties of the plants. In our study, increased contents of chlorophyll pigments in chickpea leaves were observed in co-inoculated plants with *M. ciceri* and *B. subtilis*. Similar results were obtained by Heidari and Golpayegani (2012) for *Ocimum basilicum* grown under water stress, where a combined inoculation of *Pseudomonas* sp. and *Bacillus lentus* in plants stimulated chlorophyll synthesis as well as photosynthetic electron transport. Abiotic stress can increase hydrogen peroxide production in plants which is associated with membrane leakage (Ahmad et al., 2012). We have observed that hydrogen peroxide concentration in leaves of tomato grown in saline soil was decreased by bacterial inoculation compared to untreated plants. It is known that stress factors increase the production and accumulation of reactive oxygen species (ROS) while endophytic bacteria colonizing plant tissue reducing H_2_O_2_ synthesis may protect the membrane lipids from peroxidation. Hashem et al. (2016) also found that *B. subtilis* isolated from *A. gerrardii* plant tissue, reduced H_2_O_2_ production under salt stress conditions. Soluble proteins protect plants under stress, which result in an improved stress tolerance and reflect the availability of nitrogen for growth and development of plants (Andrews et al., 1999). Accordingly, we also observed higher soluble leaf protein concentrations in dual inoculated chickpea tissues with *M. ciceri* and *B. subtilis* compared to the control plants. A compatible osmolyte such as proline, glycine, or betaine plays an important role in plant tolerance to stress factors through osmotic adjustment (Hashem et al., 2015). We observed an increased proline content in chickpea plants inoculated with *M. ciceri* and *B. subtilis* that resulted in stress adaptation in saline soil. A similar observation was reported by Vardharajula et al. (2011) for maize, where *Bacillus* spp. improved plant growth and tolerance to drought stress via enhanced production of proline, amino acids, and soluble sugars.

In conclusion, we found first evidence of root rot of chickpea caused by *F. solani* in saline soils of Uzbekistan. The endophytic bacterial isolates with best PGP traits were capable to reduce the infection rate of root rot in chickpea and were effective in growth stimulation and resistance to salt stress. Furthermore, the mutualistic interaction of endophytic bacteria *B. subtilis* and *M. ciceri* improved the symbiotic performance of *M. ciceri* with host plants under saline soil conditions. Our findings demonstrated that endophytic bacteria which potentially colonize root tissue are effective biological control agents of chickpea root rot but also plant growth stimulators under saline soil conditions. Such multiple plant-microbial relationships could provide promising practical approaches to increase the productivity of legumes under salt stress.

## Author contributions

DE and SW did experimental design work. DE and VS conducted experiments. AH and EA analyzed the data. DE, SW, and EA wrote the manuscript. All authors read and approved the manuscript.

### Conflict of interest statement

The authors declare that the research was conducted in the absence of any commercial or financial relationships that could be construed as a potential conflict of interest.
