# Supplementary material for: Endophytic Bacteria Improve Plant Growth, Symbiotic Performance of Chickpea (Cicer arietinum L.) and Induce Suppression of Root Rot Caused by Fusarium solani under Salt Stress
Source: Front Microbiol. 2017 Sep 28;8:1887. doi: 10.3389/fmicb.2017.01887 (PMC5625113; doi:10.3389/fmicb.2017.01887)
Supplement: Supplementary file 1 [file DataSheet1.docx]

Fig 1S.

c

b

ab

a

a

**Figure 1S.** Biological control of chickpea black root rot by selected endophytic bacterial isolates in saline soil. Bacteria were coated on pre-germinated chickpea seeds, and plants were grown under greenhouse conditions in pots containing saline soil infested with *F. solani* spores, except for the positive control in which no spores were added to the soil. Column means marked by different letters indicate significant differences based on Turkey’s HSD test at P<0.05.
